# Dichloroacetate attenuates experimental acute liver injury and reduces hepatocyte apoptosis

**DOI:** 10.3389/fphar.2026.1919667

**Published:** 2026-10-26

**Authors:** Feng Xiang, Shiwen Tong, Wenwei Yin

**Affiliations:** 1 Department of Infectious Diseases, Key Laboratory of Molecular Biology for Infectious Diseases (Ministry of Education), Institute for Viral Hepatitis, the Second Affiliated Hospital, Chongqing Medical University, Chongqing, China; 2 Department of Clinical Nutrition, The Second Affiliated Hospital, Chongqing Medical University, Chongqing, China

**Keywords:** A20, acute liver injury, apoptosis, Dichloroacetate, hepatoprotection, TNF-α

## Abstract

**Background:**

Acute liver injury (ALI) is a severe clinical condition characterized by rapid hepatic dysfunction and may progress to acute liver failure. Hepatocyte apoptosis is a critical pathological event in ALI. Dichloroacetate (DCA), an inhibitor of pyruvate dehydrogenase kinase, has been reported to regulate cellular metabolism and apoptosis; however, its role in ALI remains unclear. This study investigated the protective effects and potential mechanisms of DCA in experimental ALI.

**Methods:**

DCA was evaluated in mouse models of ALI induced by lipopolysaccharide (LPS)/d-galactosamine (D-GalN) or tumor necrosis factor-α (TNF-α)/D-GalN. Liver injury and hepatocyte apoptosis were assessed by serum transaminase activities, histopathology, and TUNEL staining. Hepatic immune cell profiles were analyzed by flow cytometry. Hepatic cytokine mRNA expression and serum cytokine levels were measured by RT-qPCR and ELISA, respectively. Apoptosis-related proteins were examined by Western blotting. Additional acetaminophen (APAP) and thioacetamide (TAA) models were used to assess DCA-mediated hepatoprotection.

**Results:**

DCA pretreatment substantially attenuated LPS/D-GalN-induced ALI, as evidenced by reduced serum transaminase levels, less severe histopathological injury, and decreased hepatocyte apoptosis. This hepatoprotective effect was not accompanied by significant changes in surface TLR4 abundance on Kupffer cells, serum inflammatory cytokine levels, or hepatic immune cell composition at the examined time points. Notably, DCA treatment was associated with reduced cleaved caspase-3 expression and increased A20 protein levels in both LPS/D-GalN- and TNF-α/D-GalN-treated mice.

**Conclusion:**

DCA attenuates experimental ALI and is associated with reduced hepatocyte apoptosis and increased A20 expression.

## 1 Introduction

Acute liver injury (ALI) is characterized by acute hepatocellular damage and impaired liver function of varying severity and, in severe cases, may progress to acute liver failure (ALF), a life-threatening condition associated with coagulopathy and hepatic encephalopathy (Maiwall et al., 2024). The pathological features of ALI commonly include hepatocellular necrosis and apoptosis. Although the predominant mode of hepatocyte death varies according to the etiology of liver injury, hepatocyte apoptosis represents an important pathological process in several forms of experimental ALI (Dara et al., 2015; Guicciardi and Gores, 2005). Once ALI progresses to ALF, therapeutic options become limited, and liver transplantation remains the most effective treatment for patients with severe and irreversible liver dysfunction (Fernández et al., 2024; Bernal et al., 2013). However, donor organ shortage, postoperative infection, graft rejection, and other limitations continue to restrict its clinical application (Jadlowiec and Taner, 2016). Therefore, identifying pharmacological strategies capable of limiting acute hepatocellular injury and preventing its progression remains an important therapeutic goal.

Co-administration of lipopolysaccharide (LPS) and d-galactosamine (D-GalN) induces severe ALI in mice and is widely used as an experimental model for investigating the mechanisms of immune-mediated hepatocellular injury and evaluating potential hepatoprotective interventions (Tunon et al., 2009). D-GalN depletes intracellular uridine nucleotides and thereby sensitizes hepatocytes to inflammatory injury (Newsome et al., 2000). In this model, LPS is recognized by Toll-like receptor 4 (TLR4) expressed on Kupffer cells (KCs), leading to activation of downstream inflammatory signaling pathways and the release of pro-inflammatory cytokines, including tumor necrosis factor-alpha (TNF-α), interleukin-6 (IL-6), and IL-1β (Ronca et al., 2025; Schwabe et al., 2006). Among these mediators, TNF-α plays a central role in hepatocellular injury. TNF-α binds to tumor necrosis factor receptor-1 (TNFR1) and tumor necrosis factor receptor-2 (TNFR2) on hepatocytes and can trigger apoptotic signaling through death receptor–mediated extrinsic pathways as well as mitochondria-dependent intrinsic pathways (van Loo and Bertrand, 2023). Thus, the LPS/D-GalN model provides a useful experimental system for examining the contribution of inflammatory signaling and hepatocyte apoptosis to ALI.

Dichloroacetate (DCA) is a well-characterized inhibitor of pyruvate dehydrogenase kinase (PDK) and has been reported to promote mitochondrial oxidative metabolism of pyruvate and modulate cellular energy metabolism under inflammatory conditions (Schoenmann et al., 2023). Previous studies have shown that DCA reduces lactate accumulation and improves organ function in systemic inflammatory conditions, including sepsis and endotoxin-induced shock (McCall et al., 2018; Mainali et al., 2021; Preiser et al., 1990). In addition, DCA has been reported to regulate apoptosis in multiple cancer cell lines, including non-small cell lung cancer, prostate cancer, and endometrial cancer, through modulation of apoptosis-related proteins such as B-cell lymphoma 2 (Bcl-2) family members and caspases (Yang and Tam, 2016; Cao et al., 2008; Wong et al., 2008). DCA has also been shown to affect macrophage polarization and the production of pro-inflammatory cytokines, including IL-1β and IL-6 (Na et al., 2020; Min et al., 2019). In the context of liver injury, DCA has been reported to attenuate hepatic necrosis induced by APAP and carbon tetrachloride (CCl_4_), thereby limiting subsequent tissue damage and inflammatory responses (Theodoratos et al., 2012; Irita et al., 1994a). An earlier study also demonstrated that DCA ameliorated liver injury in endotoxin-challenged fasted rats (Irita et al., 1994b). However, that study primarily focused on alterations in energy metabolism under fasting conditions, whereas the potential effects of DCA on inflammatory responses, hepatocyte apoptosis, and related molecular pathways in experimental ALI remain incompletely understood. Therefore, the present study aimed to investigate the hepatoprotective effects of DCA in LPS/D-GalN–induced ALI and to explore the cellular and molecular changes associated with DCA treatment.

## 2 Materials and methods

### 2.1 Reagents

DCA, LPS (*Escherichia coli*, O55:B5), D-GalN, and thioacetamide (TAA) were obtained from Sigma-Aldrich (St. Louis, MO, USA). APAP was purchased from APExBIO Technology LLC (Houston, TX, USA). Recombinant mouse TNF-α and fluorochrome-conjugated antibodies were purchased from BioLegend (San Diego, CA, USA), including PE-Cy5.5 anti-mouse F4/80 (clone BM8), FITC anti-mouse CD11b (clone M1170), APC anti-mouse Ly-6G (clone 1A8), PE anti-mouse TLR4 (clone SA15-21), PerCP-Cy5.5 anti-mouse NK1.1 (clone PK136), APC anti-mouse CD3 (clone 17A2), FITC anti-mouse CD4 (clone RM4-5), and PE-Cy7 anti-mouse CD8 (clone 53-6.7). The anti-β-actin antibody (rabbit) was purchased from Servicebio (Wuhan, China). Anti-Bcl-2 and anti-BAX antibodies were obtained from Abcam (Cambridge, UK), whereas anti-X-linked inhibitor of apoptosis protein (XIAP), anti-FLICE-like inhibitory protein (FLIP), anti-tumor necrosis factor alpha-induced protein 3 (A20), and anti-cleaved caspase-3 antibodies were purchased from Cell Signaling Technology (Danvers, MA, USA). All primary antibodies used for Western blotting were rabbit antibodies.

### 2.2 Animal experiments

Male C57BL/6J mice (6–8 weeks old) were used in this study. All animals were obtained from the Experimental Animal Center of The Second Affiliated Hospital of Chongqing Medical University. Mice were maintained under controlled conditions (20 °C–25 °C, 50% ± 5% relative humidity, and a 12-h light/dark cycle) in a barrier housing facility. During the 7-day acclimatization period, mice were provided *ad libitum* access to standard laboratory chow supplied by the institutional animal facility and water. After acclimatization, mice were allocated to the indicated experimental groups without deliberate selection based on animal characteristics. All animal procedures were approved by the Animal Care and Use Committee of The Second Affiliated Hospital of Chongqing Medical University (Approval No. IACUC-SAHCQMU-2023-0023) and were conducted in accordance with institutional guidelines. At the end of the study, mice were euthanized by carbon dioxide (CO_2_) inhalation. Mice were intraperitoneally (i.p.) co-injected with LPS (5 μg/kg) and D-GalN (400 mg/kg) to induce ALI (Jiang et al., 2005). The TNF-α/D-GalN-induced liver injury model was established by i.p. co-injection of 0.4 µg/mouse TNF-α and 400 mg/kg D-GalN (Leist et al., 1995). DCA was administered by i.p. injection at a dose of 300 mg/kg 0.5 h before ALI induction. The same volume of sterile PBS was used as the vehicle control for the mice. In the survival rate experiment, mice were administered different doses of DCA (100 mg/kg, 200 mg/kg, 300 mg/kg) 0.5 h before establishing the LPS/D-GalN model, with 36 h as the observation endpoint. To establish the APAP-induced liver injury model, mice were fasted for 16 h with free access to water and then i.p. injected with APAP at a dose of 300 mg/kg or 500 mg/kg (Mossanen and Tacke, 2015). Samples were collected at the indicated time points after APAP administration. In addition, TAA-induced liver injury was established by i.p. injection of TAA (500 mg/kg), and samples were collected 24 h later (Shmarakov et al., 2014). The doses of LPS/D-GalN, TNF-α/D-GalN, APAP, and TAA were selected based on established murine liver injury models reported previously, as cited above. For DCA, doses of 100, 200, and 300 mg/kg were evaluated in a dose-response survival experiment in the present study, and 300 mg/kg was selected for subsequent experiments because it produced the greatest survival benefit.

### 2.3 Biochemical analysis

Blood samples were collected 6 h after LPS/D-GalN or TNF-α/D-GalN challenge and 24 h after APAP (300 mg/kg) or TAA administration in the main experiments. For the APAP post-treatment experiment, blood samples were collected 24 h after APAP administration. For the APAP time-course experiment, blood samples were collected at 3, 6, 12, and 24 h after APAP administration, whereas in the severe APAP model (500 mg/kg), blood samples were collected at 6 h. The samples were allowed to clot at room temperature for 0.5 h and then centrifuged at 4,000 rpm for 10 min to obtain serum. Serum alanine aminotransferase (ALT) and aspartate aminotransferase (AST) activities were measured using commercial microplate assay kits obtained from Nanjing Jiancheng Bioengineering Institute (Nanjing, China). Absorbance was measured at 505 nm, and ALT and AST activities were calculated from the corresponding calibration curves according to the manufacturer’s instructions. Serum samples were diluted 5- to 30-fold with normal saline, as required, to ensure that the measured values fell within the assay range. Samples exceeding the assay range were re-diluted at an appropriate factor and reanalyzed, and the final ALT and AST activities were corrected for the corresponding dilution factors. Serum lactate dehydrogenase (LDH) and glutathione (GSH) levels were measured using commercial assay kits from the same manufacturer and calculated according to the formulas provided in the respective kit instructions.

### 2.4 Histology and TUNEL assay

Liver tissues were harvested 6 h after induction of ALI and fixed in 10% neutral buffered formalin, followed by dehydration, paraffin embedding, and sectioning at 5 µm. The sections were stained with hematoxylin and eosin (H&E) and subsequently mounted. Histological liver injury was evaluated using a semi-quantitative grading system adapted from Suzuki’s criteria (Yang et al., 2023; Suzuki et al., 1993), with modifications appropriate for the present acute liver injury models. Injury was graded on a scale of 0–4: 0, no discernible injury; 1, slight immune cell infiltration; 2, hepatocyte ballooning/degeneration; 3, loss of hepatic structure with increased apoptotic bodies or massive immune cell infiltration; and 4, massive sinusoidal congestion with loss of hepatic structure. For each mouse, 3–5 randomly selected non-overlapping fields were scored, and the mean score was used for statistical analysis. Hepatocyte apoptosis was assessed using a terminal deoxynucleotidyl transferase-mediated dUTP nick-end labeling (TUNEL) apoptosis detection kit from Servicebio (Wuhan, China) according to the manufacturer’s instructions. Histological scoring and TUNEL assessment were performed using coded samples by an investigator blinded to group allocation.

### 2.5 Cytokine assay

Serum levels of TNF-α, IL-6, IL-1β, and IL-10 were measured 2 h after model induction using mouse precoated ELISA kits obtained from DAKEWE (Shenzhen, China) according to the manufacturer’s instructions. The kit-provided recombinant cytokine standards were reconstituted and serially twofold diluted with the supplied dilution buffer to generate standard curves. The standard concentration ranges were 15.6–1000 pg/mL for TNF-α and IL-6, 31.3–2000 pg/mL for IL-1β, and 62.5–4000 pg/mL for IL-10. Serum samples were typically diluted 5- to 10-fold, as appropriate, using the sample dilution buffer supplied with the kits to ensure that measured concentrations fell within the corresponding standard-curve ranges. Absorbance was measured at 450 nm, and cytokine concentrations in unknown samples were determined by interpolation from the corresponding standard curves and corrected for the appropriate dilution factors. Samples with concentrations exceeding the upper limit of the standard curve were further diluted and reassayed.

### 2.6 Purification of liver mononuclear cells

Liver mononuclear cells (LMNCs) were isolated as previously described with minor modifications (Koda et al., 2021). After removal of the gallbladder, liver tissues were homogenized and passed through a 200-mesh strainer to remove debris. The filtrates were centrifuged at 50 *g* for 5 min at 4 °C, and the supernatants were subsequently collected and centrifuged at 2,000 rpm for 10 min. Cell pellets were resuspended in 2 mL of 40% Percoll and subjected to density-gradient centrifugation at 1,260 × g for 20 min at room temperature. After red blood cell lysis and washing with staining buffer, liver mononuclear cells were isolated for subsequent analyses.

### 2.7 Flow cytometry

Isolated LMNCs were blocked with rat serum for 10 min and then incubated with fluorochrome-conjugated antibodies for 30 min at 4 °C in the dark. After washing with staining buffer, cells were resuspended in staining buffer and analyzed using a Beckman Coulter flow cytometer (CA, USA). Flow cytometry data were analyzed using FlowJo software (version 10.0).

### 2.8 RT-qPCR

Total RNA was extracted from liver tissues using commercial reagents obtained from TaKaRa Bio Inc. (Shiga, Japan) according to the manufacturer’s instructions. RNA concentration and purity were determined using a spectrophotometer. Genomic DNA was removed and complementary DNA was synthesized by reverse transcription. Quantitative reverse transcription polymerase chain reaction (RT-qPCR) was performed using a SYBR Green-based detection system. Relative mRNA expression levels were calculated using the comparative 2^−ΔΔCt method with β-actin as the internal reference gene (Schmittgen and Livak, 2008). No external standard curve was used for qPCR quantification. Primer sequences are listed in Table 1.

**TABLE 1:** Primer sequences used for RT-qPCR analysis of liver tissue.

Gene name	Sequence (5′→ 3′)
TNF-α	F: GCC​ACC​ACG​CTC​TTC​TGT​CT
	R: GGT​CTG​GGC​CAT​AGA​ACT​GAT​G
IL-6	F: TGA​CGG​CAT​TTG​AAG​GGG​TC
	R: GCA​GCG​GTC​TGA​AGG​TTT​GA
IL-1β	F: GCA​ACT​GTT​CCT​GAA​CTC​AAC​T
	R: ATC​TTT​TGG​GGT​CCG​TCA​ACT
IL-10	F: GTC​CCT​GCT​GGA​GGA​CCT​TA
	R: GAT​GGC​TGG​ACT​GTG​GTT​CT
β-actin	F: ATT​CAT​GCG​GCC​AAA​GAG​GA
	R: CCG​TGC​GAG​TCA​GAG​AAA​GA

### 2.9 Western blot analysis

Total protein from mouse liver tissues was extracted using a whole-protein extraction kit (KGI Bio, China). Equal amounts of protein were mixed with SDS-PAGE loading buffer, boiled, and stored at −20 °C. Proteins were separated by SDS-PAGE and transferred onto PVDF membranes. After blocking with 5% non-fat milk for 1 h, membranes were incubated with primary antibodies (1:1000 dilution) at 4 °C overnight, followed by incubation with secondary antibodies (1:10,000 dilution) for 1 h at room temperature. Protein bands were visualized using a chemiluminescence imaging system (Bio-Rad, USA), and band intensities were quantified using ImageJ software and normalized to the corresponding β-actin signal.

### 2.10 Statistical analysis

Statistical analyses were performed using SPSS 26.0 and GraphPad Prism 10. Data distribution and variance were assessed before analysis. Two-group comparisons were performed using an unpaired Student’s t-test when parametric assumptions were met. For three or more independent groups, one-way ANOVA was followed by Dunnett’s test when multiple groups were compared with a common reference group, or by Šídák’s test for selected pairwise comparisons. Time-course experiments were analyzed using two-way ANOVA followed by Šídák’s multiple-comparison test. ALT and LDH time-course data were log10-transformed before analysis and are presented on the original scale. Histological injury scores were analyzed using nonparametric methods because of their ordinal nature, with the exact Mann–Whitney U test used for the primary comparison between the injury group and the corresponding DCA-treated injury group. Survival curves were compared using the log-rank test. Nonparametric alternatives were used when parametric assumptions were not satisfied. A *p* value <0.05 was considered statistically significant.

## 3 Results

### 3.1 DCA pretreatment attenuates LPS/D-GalN-induced ALI

Combined administration of LPS and D-GalN rapidly provokes fulminant hepatic injury in mice, resulting in high mortality within a short timeframe (Tunon et al., 2009). To investigate the potential efficacy of DCA against LPS/D-GalN–induced ALI, we conducted a survival analysis. Mice received DCA pretreatment at doses of 100 mg/kg, 200 mg/kg, or 300 mg/kg, followed by ALI induction. Survival was monitored and documented at 3 h intervals for up to 36 h. The results showed that all mice in the LPS/D-GalN group succumbed within 12 h following ALI induction. Pretreatment with 100 mg/kg DCA failed to confer any survival benefit. At the 36 h endpoint, survival rates in the 200 mg/kg and 300 mg/kg DCA groups were 40% and 80%, respectively, indicating that DCA improved survival in LPS/D-GalN–treated mice in a dose-dependent manner (Figure 1A).

**FIGURE 1:**
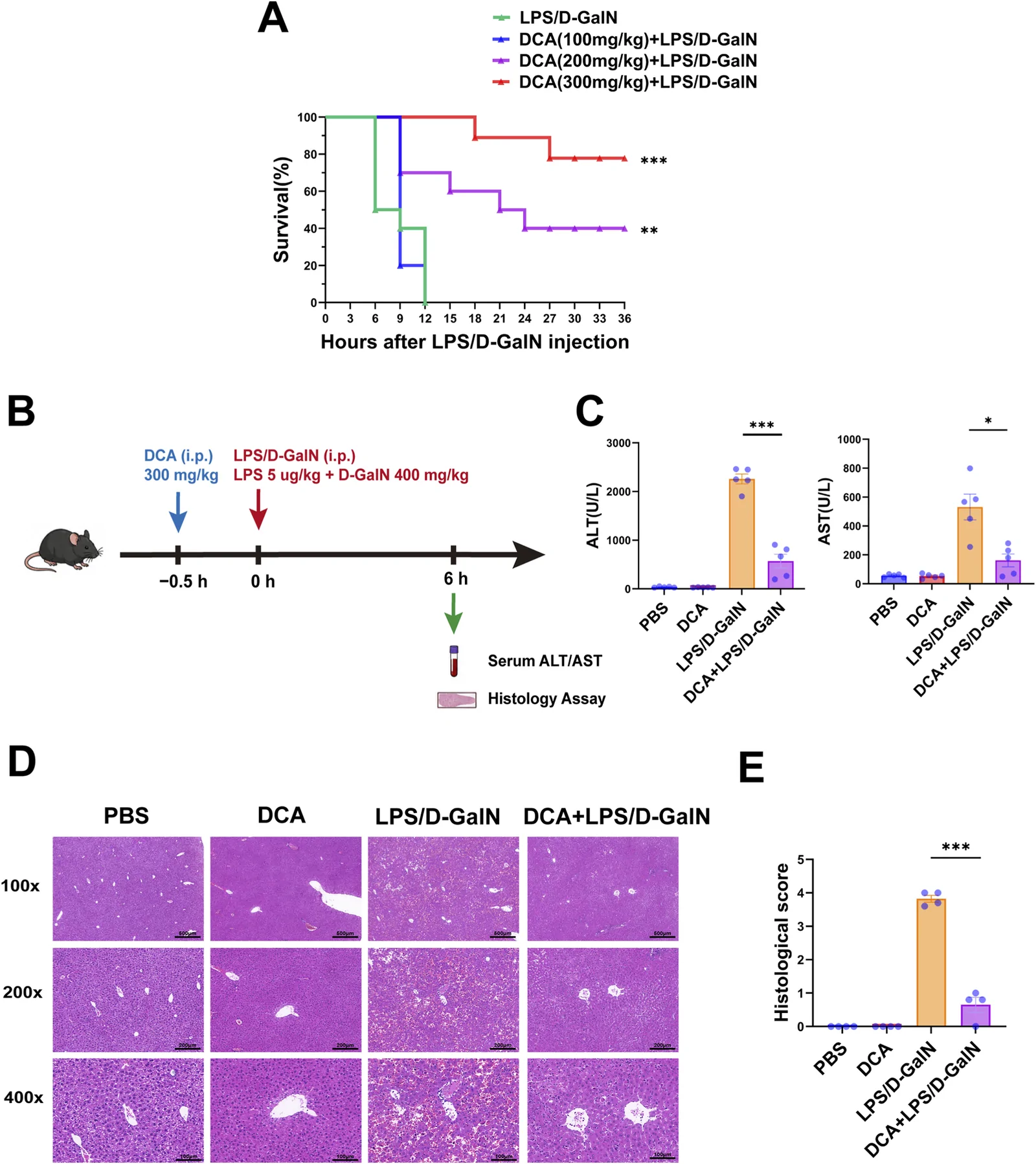
DCA pretreatment attenuates LPS/D-GalN-induced acute liver injury in mice C57BL/6J mice were intraperitoneally administered DCA (300 mg/kg) 0.5 h prior to LPS (5 μg/kg) and D-GalN (400 mg/kg) challenge. Blood and liver tissues were collected 6 h after stimulation. **(A)** Survival curves of LPS/D-GalN-treated mice pretreated with different doses of DCA. **(B)** Schematic diagram of the animal experimental design (Illustrations adapted from DBCLS TogoTV, licensed under CC BY 4.0.). **(C)** Serum alanine aminotransferase (ALT) and aspartate aminotransferase (AST) levels. **(D)** Representative liver histopathology assessed by hematoxylin and eosin (H&E) staining. **(E)** Histological injury scores of liver sections. Survival data are presented as Kaplan–Meier curves (n = 10; panel **(A)**). Other data are presented as mean ± SEM (n = 4–5). Statistical significance is indicated as *p < 0.05, **p < 0.01, and ***p < 0.001.

Based on these findings, a dose of 300 mg/kg DCA was selected for subsequent experiments. Mice were intraperitoneally administered DCA 0.5 h before LPS/D-GalN challenge, and blood and liver tissues were collected 6 h after ALI induction for further analyses (Figure 1B). The results indicated that DCA pretreatment significantly reduced serum ALT and AST levels in LPS/D-GalN-treated mice (Figure 1C). Histopathological examination revealed that livers from LPS/D-GalN–treated mice exhibited severe disruption of hepatic architecture accompanied by extensive hemorrhage and hepatocellular injury, whereas these pathological alterations were markedly attenuated by DCA pretreatment (Figure 1D). Consistently, histological scoring demonstrated that DCA-treated mice showed significantly lower liver injury scores than those in the LPS/D-GalN group (Figure 1E). These findings indicate that DCA effectively alleviates LPS/D-GalN–induced ALI.

### 3.2 DCA does not alter surface TLR4 abundance on Kupffer cells or hepatic immune cell composition

In the LPS/D-GalN–induced ALI model, activation of KCs through surface TLR4 is considered a key mechanistic event (Jiang et al., 2005; El Kasmi et al., 2012). We therefore assessed surface TLR4 abundance on KCs by flow cytometry. Surface TLR4 abundance on KCs, as measured by mean fluorescence intensity (MFI), was comparable between the LPS/D-GalN and DCA-treated groups (Figure 2A), indicating that DCA treatment did not significantly alter surface TLR4 abundance on KCs at the examined time point. We next examined hepatic immune cell composition. Flow cytometric analysis showed that the proportions of KCs and neutrophils were similar between the model and DCA-treated groups (Figures 2B,C). In addition, the relative proportions of lymphocyte subsets, including total T cells, natural killer T (NKT) cells, natural killer (NK) cells, CD4^+^ T cells, and CD8^+^ T cells, were not significantly affected by DCA treatment (Figures 2D,E). Collectively, these results indicate that the hepatoprotective effect of DCA in LPS/D-GalN–induced liver injury was not associated with detectable changes in surface TLR4 abundance on KCs or hepatic immune cell composition at the examined time point.

**FIGURE 2:**
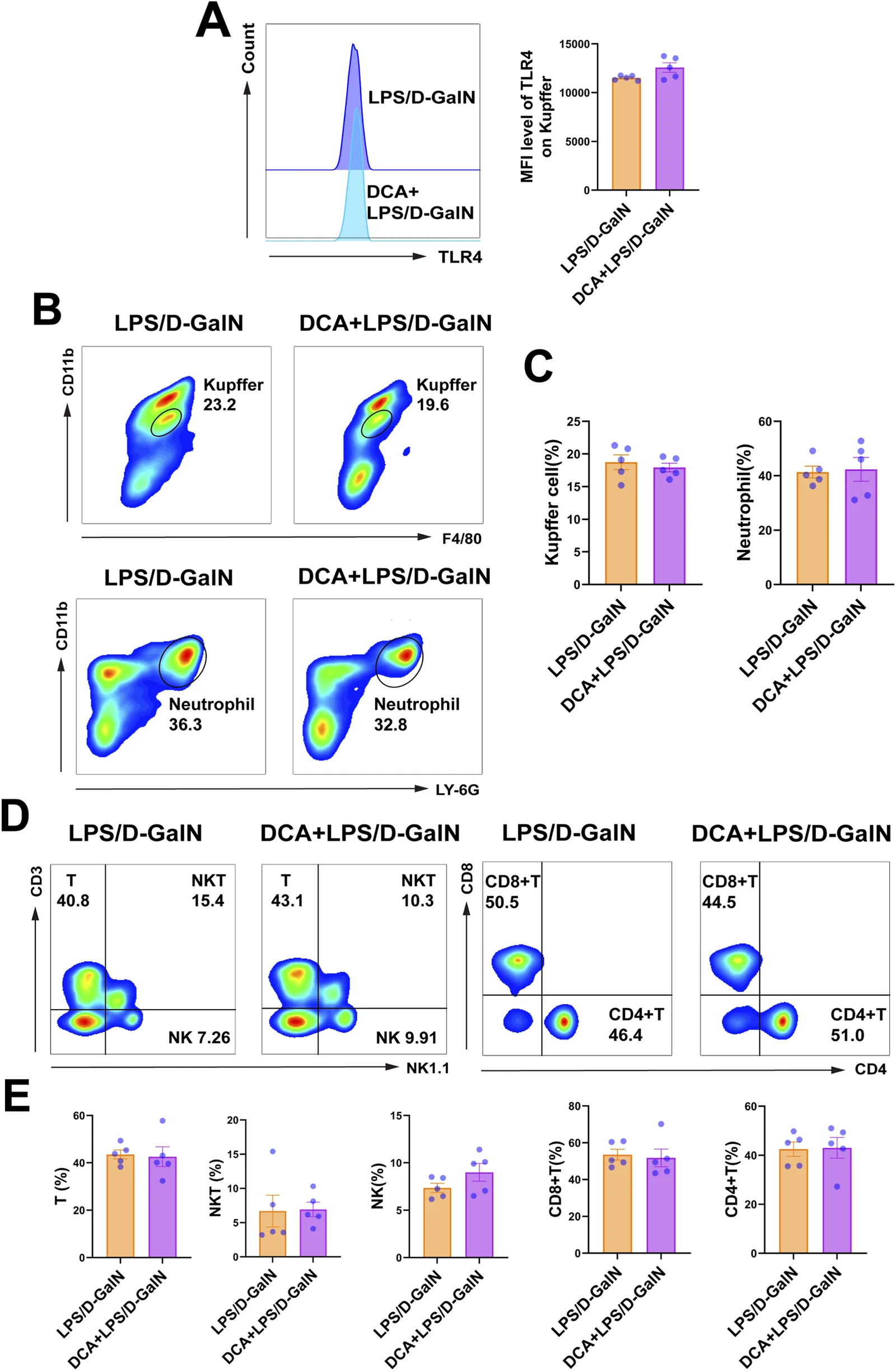
DCA does not alter surface TLR4 abundance on Kupffer cells or hepatic immune cell composition Mice were treated with DCA followed by LPS/D-GalN challenge. Liver mononuclear cells were isolated 6 h later for flow cytometric analysis. **(A)** Representative histograms of surface TLR4 abundance on Kupffer cells and corresponding mean fluorescence intensity (MFI) quantification. **(B)** Representative flow cytometry plots of Kupffer cells and neutrophils. **(C)** Percentage of Kupffer cells and neutrophils. **(D)** Representative flow cytometry plots of lymphocyte subsets, including T, NKT, and NK cells, as well as CD4^+^ and CD8^+^ T cells (gated from T cells). **(E)** Percentage of the indicated lymphocyte subsets. Data are expressed as the mean ± SEM (n = 5).

### 3.3 DCA does not alter inflammatory cytokine responses following LPS/D-GalN challenge

Previous studies have identified inflammatory cytokines as critical mediators of LPS/D-GalN–induced acute liver injury (Yang et al., 2016). To determine whether the hepatoprotective effect of DCA is associated with modulation of the inflammatory response, hepatic cytokine mRNA expression and serum cytokine levels were examined 2 h after LPS/D-GalN challenge. No significant differences were observed in the hepatic mRNA expression levels of TNF-α, IL-6, IL-1β, or IL-10 between the DCA-treated and LPS/D-GalN groups (Figure 3A). Likewise, serum concentrations of TNF-α, IL-6, IL-1β, and IL-10 were comparable between the two groups (Figure 3B). These findings suggest that the protective effect of DCA against LPS/D-GalN–induced liver injury is unlikely to be mediated through the regulation of inflammatory cytokine responses.

**FIGURE 3:**
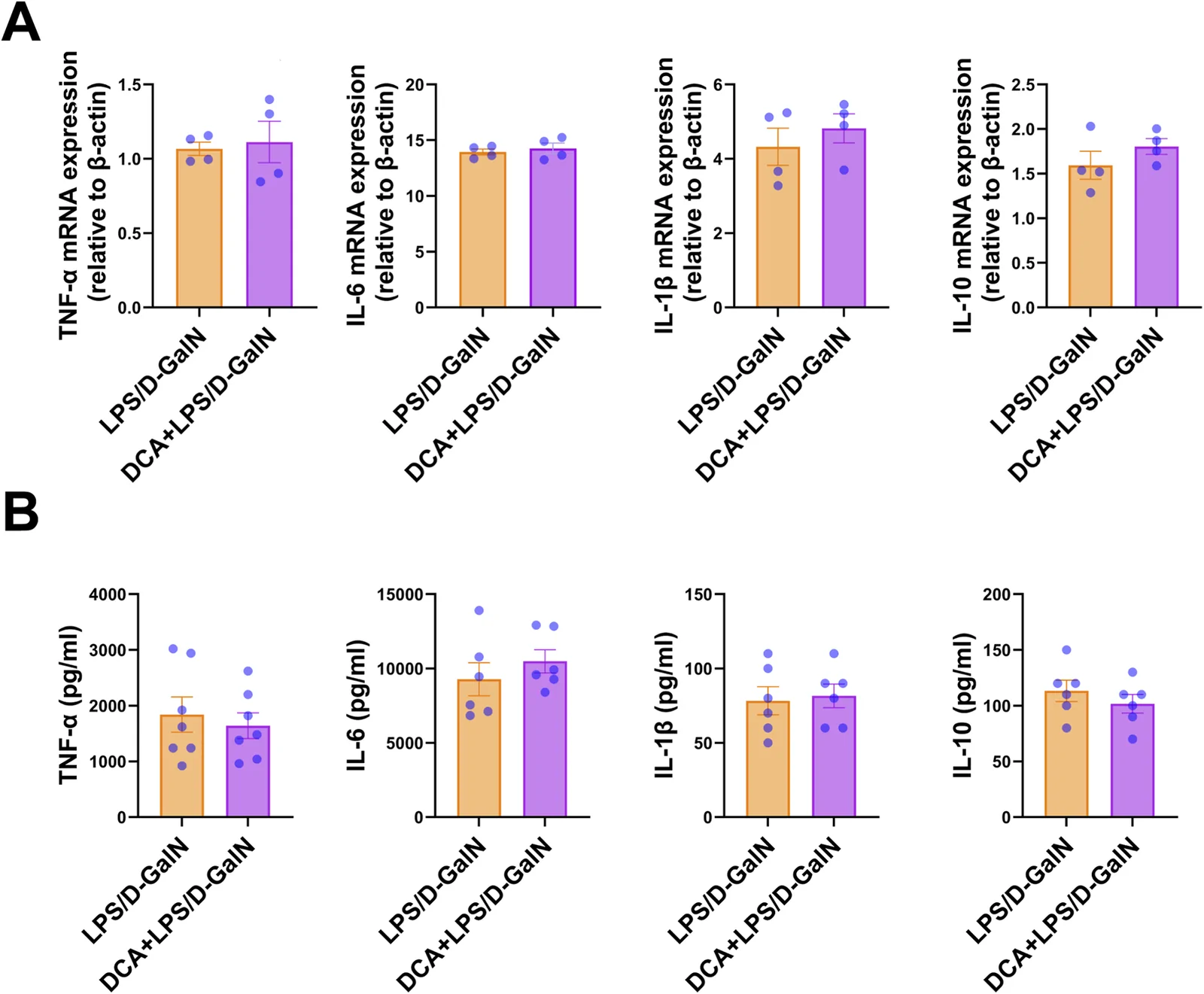
DCA does not alter inflammatory cytokine responses following LPS/D-GalN challenge Mice were treated with DCA followed by LPS/D-GalN challenge. Liver tissues and serum samples were collected 2 h after LPS/D-GalN administration. **(A)** Hepatic mRNA expression levels of TNF-α, IL-6, IL-1β, and IL-10 were determined by quantitative real-time PCR. **(B)** Serum levels of TNF-α, IL-6, IL-1β, and IL-10 were measured by ELISA. Data are presented as mean ± SEM (n = 4–6).

### 3.4 DCA alleviates LPS/D-GalN–induced hepatocellular apoptosis

TNF-α–mediated receptor signaling is a key driver of hepatocyte apoptosis in LPS/D-GalN–induced liver injury (Leist et al., 1995). Given that DCA did not affect inflammatory cytokine responses in the LPS/D-GalN model, we next investigated whether its protective effect is associated with modulation of hepatocyte apoptosis. Accordingly, TUNEL fluorescence staining was performed to assess the extent of hepatocyte apoptosis. The number of TUNEL-positive hepatocytes (green fluorescence) was markedly reduced in the DCA + LPS/D-GalN group compared with the LPS/D-GalN group (Figure 4A), indicating that DCA effectively attenuates hepatocyte apoptosis. Cleaved caspase-3 represents the activated form of caspase-3 and serves as a hallmark executioner marker of apoptosis (Kurokawa and Kornbluth, 2009). To further confirm this observation, we examined the expression of cleaved caspase-3 by Western blot. DCA treatment markedly reduced the expression of cleaved caspase-3 in liver tissues (Figure 4B). Consistently, quantitative analysis of cleaved caspase-3 normalized to β-actin showed a significant decrease in the DCA-treated group (Figure 4C).

**FIGURE 4:**
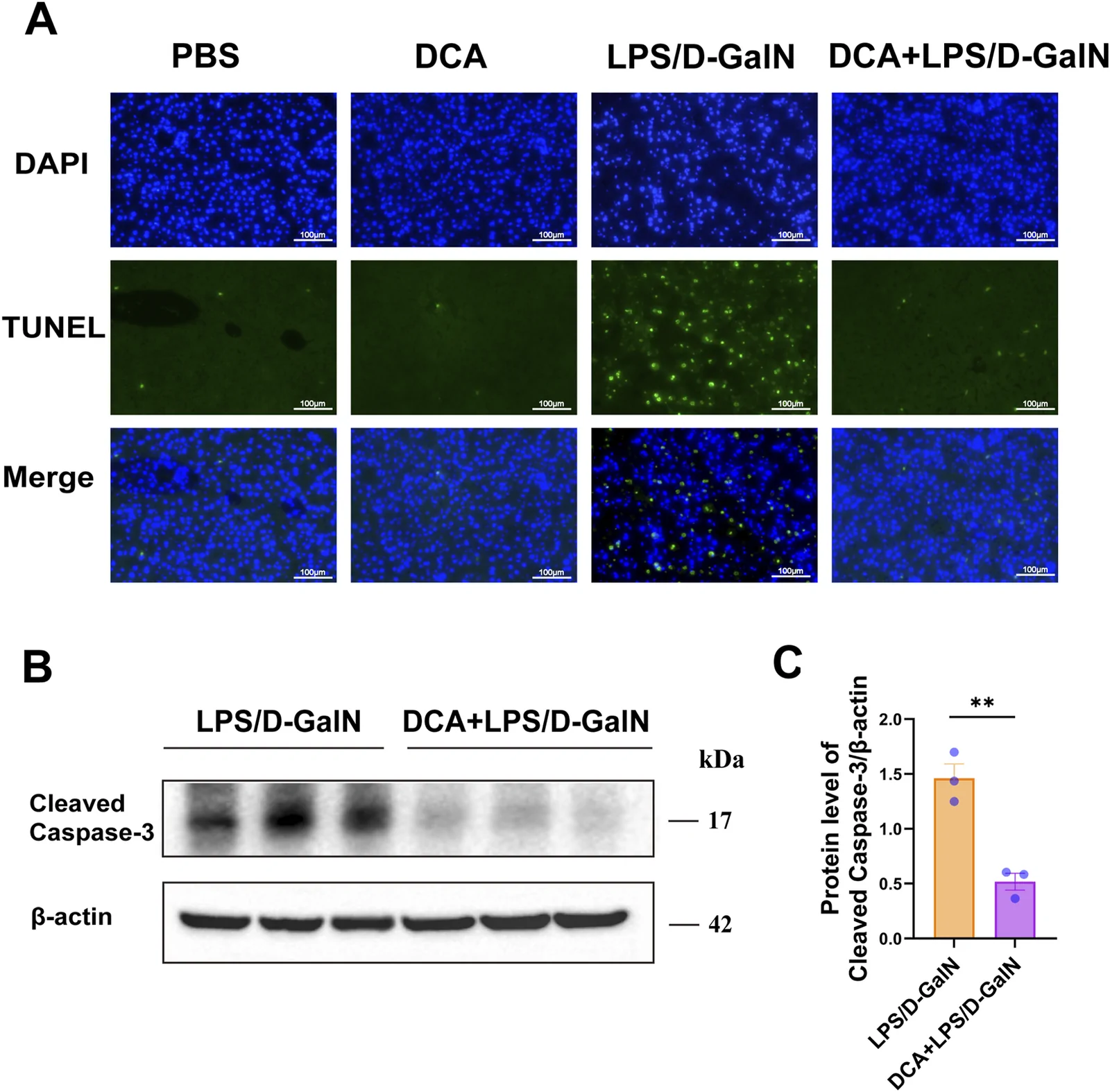
DCA alleviates LPS/D-GalN–induced hepatocellular apoptosis Mice were pretreated with DCA 0.5 h before LPS/D-GalN challenge. Liver tissues were collected 6 h later for TUNEL and Western blot analyses. **(A)** Representative TUNEL staining of liver sections (apoptotic cells are shown in green). **(B)** Western blot analysis of cleaved caspase-3 expression in liver tissues. **(C)** Quantification of cleaved caspase-3 expression normalized to β-actin. Data are presented as mean ± SEM (n = 3–5). Statistical significance is indicated as *p < 0.05, **p < 0.01, and ***p < 0.001.

### 3.5 DCA protects against TNF-α/D-GalN–induced ALI

To further examine whether the hepatoprotective effect of DCA is retained when liver injury is triggered by exogenous TNF-α, we established a TNF-α/D-GalN–induced ALI model. Mice were pretreated with DCA for 0.5 h, followed by i.p. injection of recombinant TNF-α (0.4 µg/mouse) and D-GalN (400 mg/kg). At 6 h, liver injury was evaluated based on serum transaminase levels and histological analysis by H&E staining. The results showed that serum ALT and AST activities were markedly reduced by DCA pretreatment in TNF-α/D-GalN–treated mice (Figure 5A). Histopathological examination revealed that DCA markedly attenuated TNF-α/D-GalN–induced hepatic architectural disruption and sinusoidal hemorrhage, which was further confirmed by significantly lower histological injury scores in DCA-treated mice compared with the TNF-α/D-GalN group (Figures 5B,C). TNF-α/D-GalN treatment was associated with abundant TUNEL-positive cells (green fluorescence), whereas fewer TUNEL-positive cells were observed following DCA pretreatment (Figure 5D). Western blot analysis further demonstrated that DCA pretreatment significantly reduced hepatic cleaved caspase-3 expression in TNF-α/D-GalN–treated mice (Figures 5E,F). These findings support an association between DCA treatment and reduced hepatocyte apoptotic injury in the TNF-α/D-GalN model.

**FIGURE 5:**
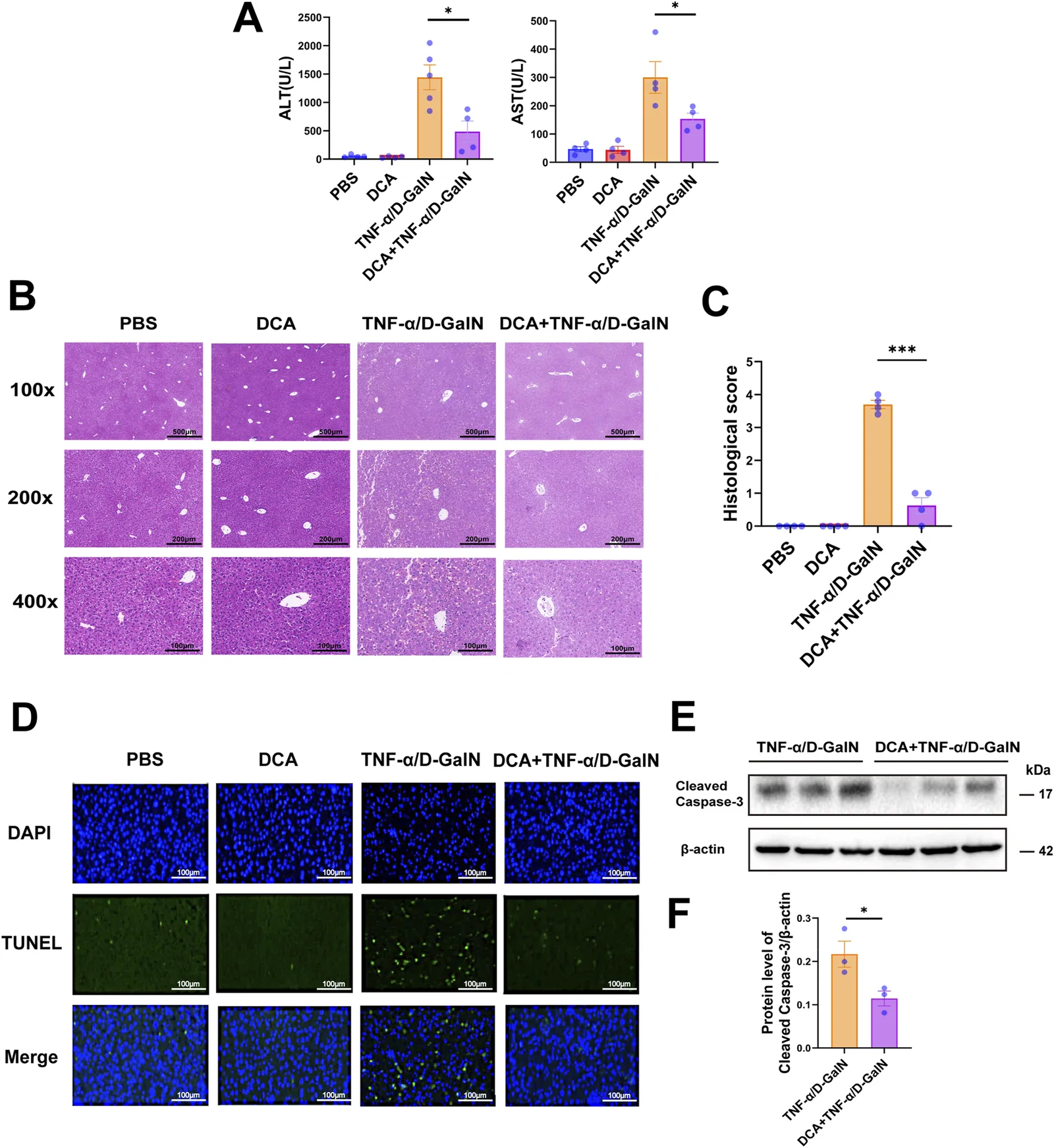
DCA protects against TNF-α/D-GalN–induced acute liver injury Mice were pretreated with DCA (300 mg/kg, i.p.) 0.5 h prior to TNF-α (0.4 μg/mouse) and D-GalN (400 mg/kg) challenge. Blood and liver samples were collected 6 h after challenge. **(A)** Serum ALT and AST levels. **(B)** Representative liver histopathology assessed by H&E staining. **(C)** Histological score of liver sections. **(D)** Representative TUNEL staining of liver sections (apoptotic cells are shown in green). **(E)** Western blot analysis of cleaved caspase-3 expression in liver tissues. **(F)** Quantification of cleaved caspase-3 expression normalized to β-actin. Data are presented as mean ± SEM (n = 3–5). Statistical significance is indicated as *p < 0.05, **p < 0.01, and ***p < 0.001.

### 3.6 DCA treatment is associated with increased A20 expression in LPS/D-GalN and TNF-α/D-GalN models

To further examine molecular changes associated with reduced apoptotic injury following DCA treatment, we examined the expression of key apoptosis-related proteins in both LPS/D-GalN and TNF-α/D-GalN models. In the LPS/D-GalN model, Western blot analysis showed that DCA treatment did not significantly alter the expression of FLIP, XIAP, Bcl-2, or BAX. In contrast, A20 protein abundance was significantly higher in the DCA-treated group compared with the LPS/D-GalN group (Figures 6A,B). Similarly, in the TNF-α/D-GalN model, DCA treatment did not affect the expression levels of FLIP, XIAP, Bcl-2, or BAX, whereas A20 protein abundance was significantly higher in the DCA-treated group (Figures 6C,D). These findings demonstrate that increased A20 protein abundance is associated with DCA treatment under both LPS/D-GalN- and TNF-α/D-GalN-induced injury conditions.

**FIGURE 6:**
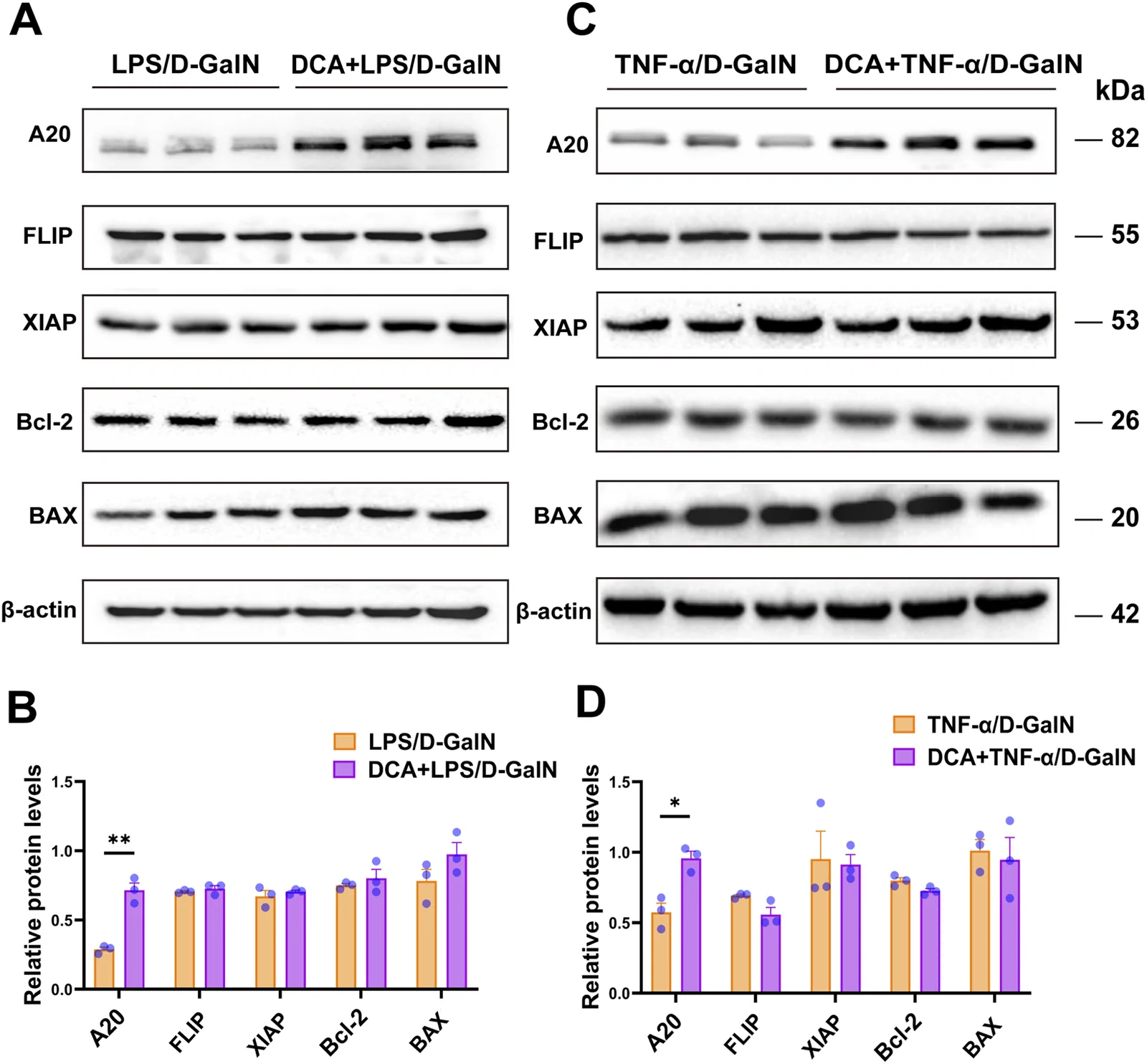
DCA treatment is associated with increased A20 expression in LPS/D-GalN and TNF-α/D-GalN models Mice were pretreated with DCA followed by LPS/D-GalN or TNF-α/D-GalN challenge. Liver tissues were collected 6 h later for analysis. **(A)** Representative Western blot analysis of A20, FLIP, XIAP, Bcl-2, BAX, and β-actin in the LPS/D-GalN model. **(B)** Densitometric quantification of the indicated proteins normalized to β-actin. **(C)** Representative Western blot analysis of A20, FLIP, XIAP, Bcl-2, BAX, and β-actin in the TNF-α/D-GalN model. **(D)** Densitometric quantification of the indicated proteins normalized to β-actin. Data are presented as mean ± SEM (n = 3). Statistical significance is indicated as *p < 0.05, **p < 0.01, and ***p < 0.001.

### 3.7 DCA attenuates liver injury in APAP- and TAA-Induced models

To further evaluate whether the hepatoprotective effect of DCA extends beyond the LPS/D-GalN and TNF-α/D-GalN models, we next examined its effects in APAP- and TAA-induced liver injury models. In the APAP-induced liver injury model, mice were pretreated with DCA (300 mg/kg, i.p.) 0.5 h prior to APAP administration (300 mg/kg, i.p.). DCA pretreatment markedly reduced serum ALT and AST activities compared with the APAP group (Figure 7A). Histopathological analysis further demonstrated that DCA alleviated APAP-induced hepatic hemorrhage and tissue injury, which was accompanied by significantly lower histological injury scores compared with those of APAP-treated mice (Figures 7B,C). To determine whether DCA retains hepatoprotective activity when administered after APAP challenge, DCA (300 mg/kg, i.p.) was administered at 0.5, 1.5, and 12 h after APAP administration. Administration of DCA at 0.5 h after APAP challenge resulted in a marked numerical reduction in serum ALT levels, although the difference did not reach statistical significance after multiple-comparison correction. In contrast, treatment at 1.5 h or 12 h did not produce an apparent reduction in serum ALT levels (Supplementary Figure S1A). In addition, DCA pretreatment also significantly attenuated liver injury induced by the higher-dose APAP model (500 mg/kg) (Supplementary Figure S1B). Time-course analysis further showed that DCA pretreatment significantly reduced serum ALT and lactate dehydrogenase (LDH) levels at 3, 6, 12, and 24 h after APAP administration (Supplementary Figure S1C). Moreover, DCA pretreatment increased serum glutathione (GSH) levels in APAP-treated mice, although post-treatment at 0.5 h did not increase GSH levels (Supplementary Figure S1D), suggesting that maintenance of GSH may contribute to the protective effect of DCA against APAP-induced liver injury.

**FIGURE 7:**
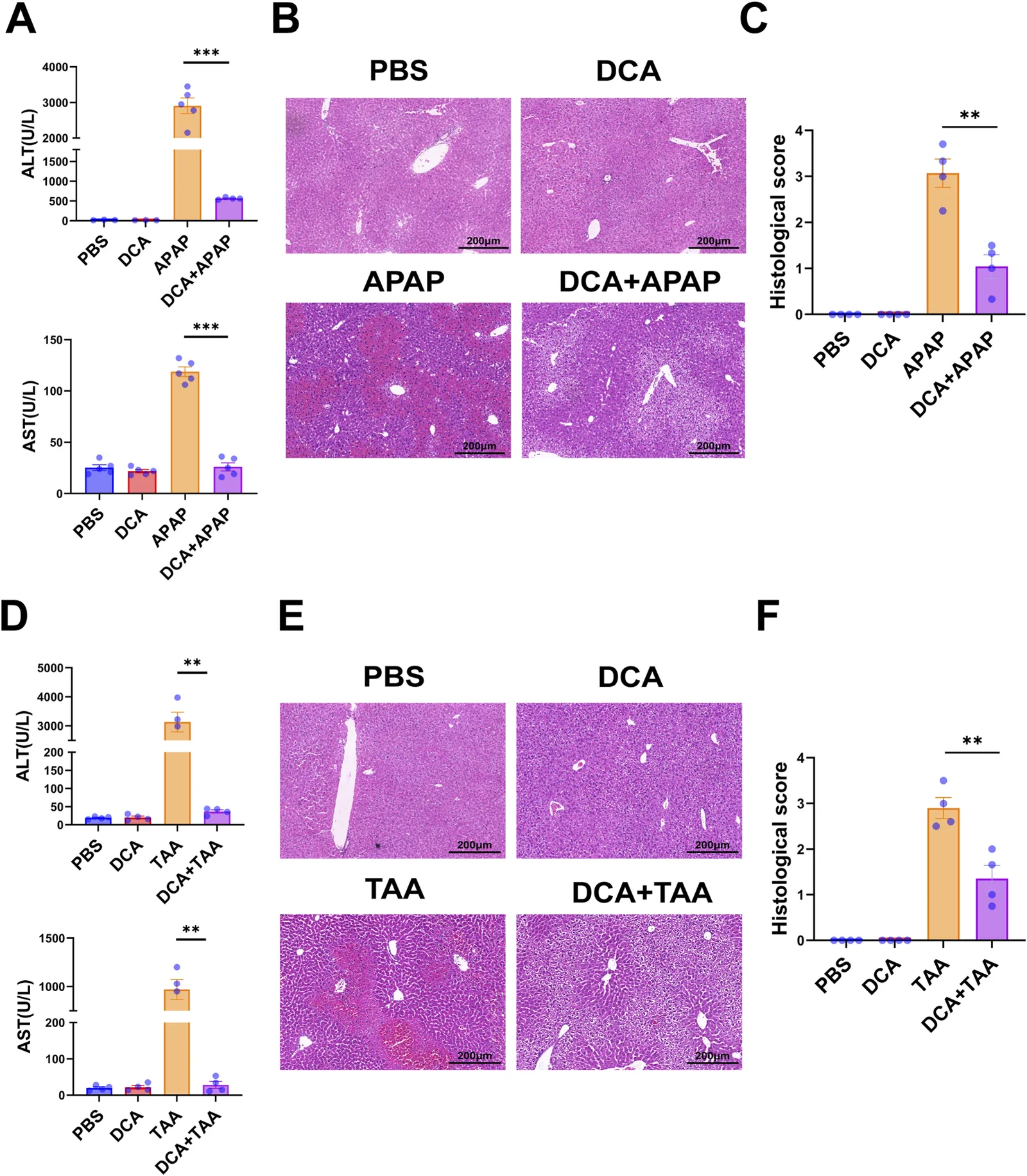
DCA attenuates liver injury in APAP- and TAA-induced models Mice were pretreated with DCA (300 mg/kg, i.p.) 0.5 h prior to APAP (300 mg/kg, i.p.) or TAA (500 mg/kg, i.p.) challenge. Blood and liver tissues were collected 24 h after challenge. **(A)** Serum ALT and AST levels in the APAP model. **(B)** Representative liver histopathology assessed by H&E staining in the APAP model. **(C)** Histological score of liver sections in the APAP model. **(D)** Serum ALT and AST levels in the TAA model. **(E)** Representative H&E staining of liver sections in the TAA model. **(F)** Histological score of liver sections. Data are presented as mean ± SEM (n = 3-5). Statistical significance is indicated as *p < 0.05, **p < 0.01, and ***p < 0.001.

TAA is a hepatotoxic agent widely used to induce experimental liver injury through oxidative stress, hepatocyte necrosis, and inflammatory responses (Ezhilarasan, 2023). Similarly, mice were pretreated with DCA (300 mg/kg, i.p.) 0.5 h prior to TAA administration (500 mg/kg, i.p.). At 24 h after challenge, DCA pretreatment markedly reversed the elevation of serum ALT and AST levels induced by TAA (Figure 7D). Histopathological examination further showed that DCA alleviated hepatic hemorrhage and structural disruption in TAA-treated mice, which was further supported by a significant reduction in histological injury scores (Figures 7E,F).

Taken together, these findings suggest that DCA confers broad hepatoprotection across multiple experimental models of ALI.

## 4 Discussion

Our findings indicate that DCA confers significant protection against LPS/D-GalN–induced ALI, as reflected by improved survival and attenuation of biochemical and histopathological liver injury. This hepatoprotective effect was not accompanied by detectable changes in surface TLR4 abundance on Kupffer cells, serum inflammatory cytokine levels, or hepatic immune cell composition at the examined time points. DCA treatment was also associated with reduced hepatocyte apoptosis and lower cleaved caspase-3 expression in both LPS/D-GalN–and TNF-α/D-GalN–treated mice. In parallel, higher A20 protein abundance was consistently observed in DCA-treated mice in both injury models. Together, these findings indicate that DCA-mediated hepatoprotection is associated with reduced apoptotic injury and increased A20 expression. However, the present data do not establish that suppression of apoptosis is the primary causal mechanism of DCA-mediated hepatoprotection, and the causal contribution of A20 also remains to be determined.

As the primary receptor mediating the cellular response to LPS, TLR4 plays a central role in LPS signal transduction in macrophages (Beutler, 2004; Beutler, 2000). In the liver, activation of KCs by LPS can enhance TLR4-mediated inflammatory signaling and promote the release of pro-inflammatory cytokines (Takayashiki et al., 2004; Su et al., 2000). Among these, TNF-α is a key mediator of hepatocyte injury in the LPS/D-GalN model (Josephs et al., 2000; Hamesch et al., 2015). In the present study, however, DCA treatment did not significantly alter the proportion of KCs, surface TLR4 abundance on KCs, or serum TNF-α levels at the examined time point. These findings suggest that the hepatoprotective effect of DCA is not associated with detectable changes in surface TLR4 abundance or systemic TNF-α levels under the conditions examined. Moreover, the proportions of neutrophils, T cells, NK cells, and NKT cells were comparable between the model and DCA-treated groups, indicating that DCA treatment was not accompanied by major changes in hepatic immune cell composition. Taken together, these observations suggest that the protective effect of DCA may involve mechanisms beyond measurable alterations in the examined inflammatory and immune parameters, and are consistent with a potential contribution of hepatocyte-intrinsic responses to injury.

TNF-α is considered a central mediator of hepatocyte apoptosis in the LPS/D-GalN model (Tiegs and Horst, 2022). To further determine whether DCA-mediated protection was associated with regulation of hepatocyte apoptotic signaling rather than secondary to modulation of upstream inflammatory responses, we established a TNF-α/D-GalN–induced liver injury model. Notably, DCA treatment also attenuated liver injury and reduced cleaved caspase-3 expression in TNF-α/D-GalN–treated mice. These findings support an association between DCA treatment and reduced hepatocyte apoptotic injury. However, they do not establish that suppression of apoptosis is the primary causal mechanism underlying DCA-mediated hepatoprotection. Functional intervention studies targeting apoptotic signaling will be required to determine causality.

A20 (also known as TNFAIP3) is a downstream regulator in the TNF-α/TNF-R1 pathway that functions to inhibit TNF-induced cell death (Martens and van Loo, 2020; Jaattela et al., 1996). Previous studies have shown that A20-deficient mice develop severe inflammation, exhibit hypersensitivity to both LPS and TNF, and undergo premature death (Lee et al., 2000). In addition, A20 has been reported to protect mice against LPS/D-GalN–induced hepatotoxicity (Arvelo et al., 2002). In the present study, higher A20 protein abundance was consistently observed in DCA-treated mice compared with the corresponding injury controls in both the LPS/D-GalN and TNF-α/D-GalN models. In contrast, the expression levels of other apoptosis-related proteins, including FLIP, XIAP, Bcl-2, and BAX, were not significantly altered following DCA treatment. However, unchanged total protein abundance does not exclude the potential involvement of BAX, Bcl-2, XIAP, or FLIP in apoptotic regulation, as their activity may also be regulated at post-translational or subcellular levels. The consistent association between increased A20 abundance and DCA treatment across two injury models identifies A20 as a potential molecular correlate of the hepatoprotective phenotype. Given the established role of A20 in limiting TNF-α–mediated cell-death signaling, this association is of potential biological relevance; however, the present data do not establish that A20 mediates the hepatoprotective effect of DCA. Demonstration of a causal role will require functional studies, particularly A20 loss-of-function experiments.

Interestingly, a previous study reported that post-injury administration of DCA attenuated inflammatory infiltration in the livers of starved rats subjected to LPS/D-GalN challenge, although the reduction in serum ALT levels did not reach statistical significance. In contrast, under our experimental conditions using non-fasted mice, administration of DCA 0.5 h after LPS/D-GalN challenge failed to confer significant hepatoprotective effects (data not shown). This discrepancy may be associated with differences in metabolic status, animal species, or experimental protocols between the two studies (Irita et al., 1994b). Notably, the hepatoprotective effects of DCA were not restricted to the LPS/D-GalN model. DCA also significantly attenuated APAP- and TAA-induced liver injury, suggesting that its protective effects may extend beyond endotoxin-mediated hepatic injury. Given that these models involve distinct pathogenic mechanisms, the consistent protective effects of DCA across multiple models imply that DCA may target common pathways associated with hepatocyte injury and cell death. The exploratory post-challenge APAP experiment suggested that any benefit of delayed DCA administration, if present, may be confined to an early intervention window, whereas later treatment showed no evidence of hepatoprotection. However, because the reduction observed at the earliest post-treatment time point did not reach statistical significance after multiple-comparison correction, this possibility should be interpreted cautiously and requires further validation.

Several limitations of the present study should also be acknowledged. First, although increased A20 protein abundance was reproducibly associated with DCA treatment in both injury models, we did not perform A20 knockdown or loss-of-function experiments to determine whether A20 is required for DCA-mediated hepatoprotection. Therefore, the present findings establish an association between increased A20 expression and the protective phenotype of DCA but do not demonstrate a causal relationship. Further studies using A20 loss-of-function approaches are required to define its functional contribution. Second, the current study was limited to experimental animal models, and the clinical applicability of DCA in patients with ALI remains to be further investigated. Taken together, our findings indicate that DCA attenuates experimental acute liver injury and is associated with reduced hepatocyte apoptosis and increased A20 expression.

## Data Availability

The raw data supporting the conclusions of this article will be made available by the authors, without undue reservation.

## Supplementary material

SUPPLEMENTARY FIGURE S1 Effects of DCA administered at different time points in APAP-induced liver injury Mice received DCA (300 mg/kg, i.p.) either before or after APAP challenge, as indicated for each experiment. For the standard model, mice received APAP (300 mg/kg), whereas in the severe model, APAP (500 mg/kg) was administered and serum ALT levels were measured at 6 h. **(A)** Serum ALT levels following DCA post-treatment at different time points in the APAP (300 mg/kg) model. **(B)** Serum ALT levels in the severe APAP (500 mg/kg) model with DCA pretreatment. **(C)** Serum ALT and lactate dehydrogenase (LDH) levels at 3, 6, 12, and 24 h after APAP (300 mg/kg) administration. **(D)** Serum glutathione (GSH) levels following DCA pretreatment or 0.5 h post-treatment in the APAP (300 mg/kg) model. Data are presented as mean ± SEM (n = 3–6). Statistical significance is indicated as *p < 0.05, **p < 0.01, and ***p < 0.001.

## Glossary

A20: TNF alpha-induced protein 3
ALI: acute liver injury
ALF: acute liver failure
ALT: alanine aminotransferase
APAP: acetaminophen
AST: aspartate aminotransferase
DCA: dichloroacetate
D-GalN: d-galactosamine
ELISA: enzyme-linked immunosorbent assay
FLIP: FLICE-like inhibitory protein
GSH: glutathione
H&E: hematoxylin and eosin
i.p: intraperitoneal
IL-6: interleukin-6
KCs: Kupffer cells
LDH: lactate dehydrogenase
LMNCs: liver mononuclear cells
LPS: lipopolysaccharide
MFI: mean fluorescence intensity
NK: natural killer
NKT: natural killer T
PBS: phosphate-buffered saline
PDK: pyruvate dehydrogenase kinase
RT-qPCR: quantitative reverse transcription polymerase chain reaction
TAA: thioacetamide
TLR4: toll-like receptor 4
TNF-α: tumor necrosis factor-alpha
TNFAIP3: TNF alpha-induced protein 3
TNFR1: tumor necrosis factor receptor-1
TNFR2: tumor necrosis factor receptor-2
TUNEL: terminal deoxynucleotidyl transferase-mediated dUTP nick-end labeling
XIAP: X-linked inhibitor of apoptosis protein
